# Comparing the adaptive landscape across trait types: larger QTL effect size in traits under biotic selection

**DOI:** 10.1186/1471-2148-11-60

**Published:** 2011-03-07

**Authors:** Allison M Louthan, Kathleen M Kay

**Affiliations:** 1 Program in Ecology and Department of Zoology and Physiology, University of Wyoming, Laramie, WY, USA; 2 Department of Ecology and Evolutionary Biology, University of California-Santa Cruz, Santa Cruz, CA, USA

## Abstract

**Background:**

In a spatially and temporally variable adaptive landscape, mutations operating in opposite directions and mutations of large effect should be commonly fixed due to the shifting locations of phenotypic optima. Similarly, an adaptive landscape with multiple phenotypic optima and deep valleys of low fitness between peaks will favor mutations of large effect. Traits under biotic selection should experience a more spatially and temporally variable adaptive landscape with more phenotypic optima than that experienced by traits under abiotic selection. To test this hypothesis, we assemble information from QTL mapping studies conducted in plants, comparing effect directions and effect sizes of detected QTL controlling traits putatively under abiotic selection to those controlling traits putatively under biotic selection.

**Results:**

We find no differences in the fraction of antagonistic QTL in traits under abiotic and biotic selection, suggesting similar consistency in selection pressure on these two types of traits. However, we find that QTL controlling traits under biotic selection have a larger effect size than those under abiotic selection, supporting our hypothesis that QTL of large effect are more commonly detected in traits under biotic selection than in traits under abiotic selection. For traits under both abiotic and biotic selection, we find a large number of QTL of large effect, with 10.7% of all QTLs detected controlling more than 20% of the variance in phenotype.

**Conclusion:**

These results suggest that mutations of large effect are more common in adaptive landscapes strongly determined by biotic forces, but that these types of adaptive landscapes do not result in a higher fraction of mutations acting in opposite directions. The high number of QTL of large effect detected shows that QTL of large effect are more common than predicted by the infinitesimal model of genetic adaptation.

## Background

The modern evolutionary synthesis in the early 20^th ^century resulted in competing models describing the relative distribution of effect sizes of the mutations fixed during adaptation [1]. Despite major advances in the field of genetics, there is still uncertainty about the number of genes that contribute to quantitative trait variation and the effect sizes of these genes [2], limiting our understanding of the genetic basis of adaptation. The recent widespread use of marker-assisted genetic mapping studies provides the opportunity to compare the predictions of theoretical models with empirically estimated genetic architectures [3-6].

Fisher's infinitesimal model, one of the first and most influential theories of the genetic basis of adaptation, assumes that each locus exerts a minute effect on an organism's phenotype, and that phenotypic change occurs via the fixation of beneficial small-effect mutations at these loci [7], with negative pleiotropic effects constraining effect sizes of fixed mutations [5,7,8]. In contrast, Fisher's geometric model and subsequent work from other researchers has shown that mutations of large effect are less likely to be lost due to drift than small-effect mutations, and thus, on average, we should predict mutations of intermediate effect during an adaptive walk [8]. In this model, mutations of large effect are more likely to be fixed early on in a bout of adaptation, when a population is far from an adaptive peak, with smaller-effect mutations that act to fine-tune the phenotype to an adaptive optimum predominating during later stages of adaptation [9-11].

These models assume directional selection moves a population up a static solitary peak in an adaptive landscape, disregarding that many adaptive landscapes harbor multiple or shifting phenotypic optima [5,9,12,13]. Adaptation models predict that fixation of a large-effect mutation is more likely in populations far away from an adaptive peak. In a shifting adaptive landscape harboring multiple phenotypic optima, populations are likely to spend more time farther away from an adaptive peak, because, while selection drives them toward a phenotypic optimum, this optimum continually shifts, moving them into adaptive valleys [14-16]. Therefore, when there are continual shifts in selective pressure, mutations acting in opposite directions and mutations of large effect should be more common. Mutations that act in opposite directions include cases where one mutation acts to increase a trait value, and the other decreases the trait value. While a large number of mutations acting in one direction (for example, all mutations act to increase a trait value) indicates consistent selective pressure [3], temporal or spatial variation in selective pressure will not consistently favor mutations acting in one direction, and will thus result in a large number of mutations acting in opposite directions. Similarly, in the latter case, mutations of large effect should be more common because shifts in phenotypic optima will repeatedly move populations farther from an adaptive peak, thus favoring the fixation of large-effect mutations [14-16]. Similarly, if there are a variety of phenotypic optima in the adaptive landscape separated by valleys of low fitness, large jumps in phenotype will be favored because they will allow populations to quickly cross these valleys. The initial spectrum of newly- arisen mutations' fitness effects are unlikely to vary systematically across trait types or types of adaptive landscapes. However, the fixation of mutations of large effect should be more common in the types of adaptive landscapes described above, those that shift temporally or exhibit deep valleys of low fitness between peaks.

In order to test these hypotheses, we compare the genetic architecture of traits putatively under abiotic and biotic selection. The strength and spatial configuration of selection driven by biotic interactions can change rapidly because they are contingent on the distributions and evolutionary trajectories of other organisms [17-19]. A variety of systems show strongly patchy spatial distributions of interacting species as well as temporal fluctuations in interacting species' distributions or densities, which can result in high spatial and temporal variation in the form or strength of selection [20-24]. Similarly, evolution in interacting species' populations can lead to variation in selection pressures [18,19]. To our knowledge, no studies have explicitly compared temporal or spatial variability in selection pressure by biotic and abiotic forces. However, traits under biotic selection, because they are subject to the stochastic processes that influence interacting species' distributions and abundances, are hypothesized to be more temporally and spatially variable compared to traits under abiotic selection [9,18]. Conversely, traits under abiotic selection, such as selection imposed by environmental gradients, are not affected by spatial or temporal fluctuations in other species' densities; thus, these traits should experience an adaptive landscape with less temporal variability than biotic pressures. Additionally, traits under biotic selection may experience a landscape with more adaptive peaks than those under abiotic selection; for example, traits under this type of selection could confer adaptations to one of several potential pollinator species, or one of a series of discrete anti-herbivore compound types. We predict that the hypothesized differences in the adaptive landscape of traits under biotic selection versus abiotic selection will lead to the differences in genetic architecture outlined above between these two types of traits.

With data from quantitative trait loci (QTL) mapping studies, which have been conducted on a variety of traits and organisms, we now have the information to conduct more detailed comparative studies of the genetic architecture for different types of traits. In brief, QTL mapping locates putative genes underlying a quantitative trait within a genome using a recombinant population derived from individuals that differ in both phenotype for the trait of interest and genotype at genetic markers of known linkage. In these phenotypically and genotypically variable hybrid offspring, researchers associate genotypic differences with variation in phenotype in order to pinpoint which genomic regions are associated with the measured traits. Each detected QTL comprises a gene or a set of linked genes, and the location of each QTL corresponds to the location of this gene or set of genes. Similarly, the relative effect size of a given QTL and the direction of action of each QTL is a net sum of the action of the genes located within that QTL.

Three pieces of information from QTL mapping studies can be used to infer differences in genetic architectures: (1) the direction of a given QTL, indicated by whether the allelic effects at a QTL are in line with or oppose the difference in phenotypic trait values of the parents, (2) the percentage of the parents' phenotypic variation in the trait (percent variance explained; PVE) associated with the QTL in question; and (3) the total number of QTL detected. Unfortunately, we cannot compare the total number of QTL detected due to differences in power across studies that affect the probability of detecting small-effect QTL. In contrast, the QTL sign test, which uses QTL effect directions, may be used to evaluate whether a trait evolved under consistent directional selection [3], because directional selection will limit the fixation of antagonistic QTL (QTL with effects that are in the opposite direction to parental differences for those traits). A recent review validated the use of the QTL sign test for inferring historically consistent directional selection, finding fewer antagonistic QTL in traits known to be subject to strong directional selection [4]. Finally, the PVE, or effect size, of a QTL provides an estimate of how much an allelic substitution at a QTL contributes to the parental difference for those traits. Though the vast majority of QTL mapping studies measure and report this metric for all detected QTL, effect sizes of QTL have not yet been compared across trait types.

We reviewed information from QTL studies on a variety of traits in natural and laboratory systems to evaluate differences in genetic architectures across trait types, and our study is unique in considering both QTL direction and PVE. Our particular interest is in comparing the genetic architecture of traits putatively under abiotic selection to that of traits putatively under biotic selection. We first quantify the direction of allelic affects of QTL controlling a given trait; this analysis verifies that directional selection, upon which all competing models of genetic architectures are based, is indeed occurring. This test also compares the historical consistency of directional selection in traits under abiotic versus biotic selection [4]; we hypothesize that directional selection is common in both types of traits, but that traits under biotic selection have a higher proportion of antagonistic QTL because in a rapidly shifting adaptive landscape experienced by traits under biotic selection, QTL operating to drive the population up one adaptive peak might be rapidly rendered maladaptive as the adaptive landscape shifts. In contrast, selection via abiotic factors may be less likely to vary so rapidly and unpredictably. Second, we compare QTL effect size distributions for different types of traits. We hypothesize that large effect QTL will be more common in traits under biotic selection, as compared to traits under abiotic selection, due to temporal variability in the adaptive landscape and many phenotypic optima. This finding would support the geometric model of adaptation, which predicts that large-effect QTL become fixed more often when populations are farther from an adaptive peak [9-11], as they are in a shifting adaptive landscape.

## Methods

We performed two separate literature reviews, one for the QTL sign test, and one for effect sizes. For both of these reviews, we confined our analysis to plant taxa, because many individuals can be grown in standardized greenhouse conditions, facilitating large- scale QTL mapping with enough replication to detect small-effect QTL. We excluded all commercially grown, domesticated, or artificially selected species, including crops, forage plants, forestry trees, and ornamentals, in order to eliminate species subject to strong artificial selection. We included model organisms such as *Arabisopsis thaliana, A. lyrata*, and *A. halleri *in this analysis, but we classified a subset of all organisms as "natural ecotypes," or strains not managed specifically for research purposes, such as mutagenized or engineered strains ("laboratory strains"). Our dataset does exhibit systematic bias, in that more traits mapped in natural strains were under biotic selection: 36% of natural traits were biotic, while only 16% of traits mapped in laboratory strains were biotic. Such model organisms may exhibit different genetic architectures based on a history of mutagenesis or selection; however, we believe a comparison of QTL architectures in traits under abiotic and biotic selection in these model organisms remains valid, for the following reasons: we know of no bias whereby traits under biotic selection are systematically targeted by or differentially affected by these selective or mutagenizing processes. Further, in laboratory strains, most of these selected or mutagenized ecotypes were used to map QTL controlling both abiotic and biotic traits in different studies; for example, across all studies that quantified QTL directions, 7 out of 94 traits mapped in the L*er *× Col cross were biotic, and 5 out of 97 traits mapped in the L*er *× Cvi cross were biotic. Thus, mutagenized ecotypes were not used exclusively for mapping abiotic or biotic traits. In spite of these caveats, although we included both natural and laboratory ecotypes in all subsequent analyses, we conducted all analyses presented here on a subset of data including only QTL detected in natural accessions and excluding all traits mapped in laboratory strains. Results are qualitatively similar, and any differences in statistical results from analyses confined to natural accessions are indicated in the text.

We grouped traits according to the following categories: traits putatively under selection by biotic factors, such as traits conferring pollinator attraction, disease resistance, or herbivore defense, and traits putatively under abiotic selection, which included all other floral traits and vegetative traits, such as plant height, mineral accumulation, or germination rates. Although some traits in the latter category may be under biotic selection, unless there was a stated putative biotic selection agent, we classified these as under abiotic selection.

### QTL sign test

If traits are under strong consistent directional selection, we expect that the effects of the associated QTL will be in a consistent, positive direction. In contrast, under temporally variable, inconsistent, or weak selection or drift, we expect a mix of positive and antagonistic effects. To distinguish these scenarios, we used the QTL sign test to test for deviations from neutrality within abiotic and biotic groupings [3,4]. We compiled a list of QTL effect directions for a variety of traits, as well as data on cross type (inter- v. intraspecific), mating system (selfing v. outcrossing), plant family, growth habit, and trait type (life history, morphology, and physiology) (Table 1). We included studies from Supplemental table five in Rieseberg et al.'s [4] analysis of QTL direction and also performed a Web Of Science search on May 5, 2009 with these keywords: "Topic = (QTL) Timespan = 2002-2009". We recorded the effect direction of each QTL detected in these studies, excluding QTL detected via modeling work, expression QTL (eQTL), and QTL with only epistatic effects. Though we attempted to minimize double counting of QTL for different traits, it is likely that some of the QTL reported here are non-independent, due to similar traits being mapped in different studies or different environments. In order to minimize double counting, when experiments were repeated with the same organisms, traits, and environment, we include only the most recent results. We classified QTL detected at different times, or in replicate experiments or environments (e.g., high v. low nitrogen content) as controlling only one trait if more than half of the QTL detected either (1) had the same effect direction and mapped to the same marker locus or (2) if the authors indicated in the text that the QTL were the same. If more than half of QTL identified in a study were unique to different times, environments, or experimental replicates, each treatment was classified as a different "trait". We included QTL controlling the same trait identified in both paternal and maternal genotypic lines as separate traits, because different markers in paternal and maternal lines prevented us from confirming these QTL were at the same locus.

**Table 1:** Individual traits that deviated from neutrality

species	trait	Total QTL	fraction of antagonistic QTL	p	abiotic v. biotic	natural v. laboratory	reference
*Silene vulgaris*	Calyx length	9	0.111	0.0391	Biotic	Natural	62

*Mimulus cardinalis *× *M. lewisii*	Stamen length	7	0	0.0156	Biotic	Natural	8

*Mimulus cardinalis *× *M. lewisii*	Pistil length	7	0	0.0156	Biotic	Natural	8

*Petunia axillaris axillaris *× *P. integrifolia inflata*	Length of corolla tube	6	0	0.023809524	Biotic	Natural	67

*Arabidopsis thaliana*	Leaf trichome density	15	0.2	0.0352	Biotic	Laboratory	57

*Arabidopsis thaliana*	Metabolic profile	157	0.4076	0.0251	Abiotic	Laboratory	92

*Arabidopsis thaliana*	Flowering date	15	0.2	0.0352	Abiotic	Laboratory	100

*Arabidopsis thaliana*	Bolting date	9	0.11111	0.0391	Abiotic	Laboratory	28

*Arabidopsis thaliana*	Indol-3-ylmethyl indolic glucosinolate content	6	0	0.0238	Abiotic	Laboratory	77

*Arabidopsis thaliana*	Freezing tolerance, long day conditions	9	0	0.00390625	Abiotic	Laboratory	46

Traits that deviated significantly from neutrality using equation 7, suggesting directional selection [3]. References from [Additional file 3].

To conduct the sign test on abiotic v. biotic trait groupings using this data set, we tallied QTL directions for each group of traits, summed total QTL and antagonistic QTL detected in abiotic or biotic traits to calculate a fraction of antagonistic QTL, and tested for significant deviations from neutrality using Orr's normal approximation for large sample sizes (ref. 3, eq. 7). We excluded traits for which only one QTL was detected from our statistical analyses, because the fraction must be one [4]. We conducted a G-test on the summed fraction of antagonistic QTL derived from trait groupings to test for the effect of abiotic v. biotic status, cross type, and mating system on the proportion of antagonistic QTL. In addition to testing for directional selection on groups of traits, we also tested for directional selection on individual traits using Orr's eq. 6 [3]. Finally, we used general linear models to determine if biotic v. abiotic status, cross type, or mating system were significant predictors of an individual trait's fraction of antagonistic QTL.

### QTL effect size

The second part of our analysis tested for differences in QTL effect size between traits putatively under abiotic or biotic selection. We contrasted percent of phenotypic variance explained (PVE) of QTL controlling traits under abiotic and biotic selection. We included studies from a separate literature review on Web of Science on January 9, 2009, with the search terms "Topic = (QTL AND plant)". For each trait, we report the number of QTL and the PVE of each QTL associated with the trait, as well as the same auxiliary variables as for the sign test data. In addition, for traits under biotic selection, we also reported whether the trait was associated with floral phenotype. Reported PVE values were derived from a variety of study designs, including Composite Interval Mapping (CIM) [25,26] and Multiple Interval Mapping (MIM) [27,28].

Our exclusion and binning methods were generally the same as for the QTL sign test studies. Additionally, we did not include studies that attempted to link QTL mapped in one organism with phenotypic variance in neighboring individuals or species. We only include studies that report the PVE of each individual QTL detected, and if PVE's are given only as binned values, we take the average of the bin. If the same QTL was mapped in different environments (e.g., different treatments or time periods), then the PVE of these QTL were averaged. We excluded one metabolic study that explicitly indicated their sample size was too low and recombinant inbred lines too variable to accurately detect effect size [29]. All analyses of effect size are performed on data from this data set, whereas all analyses of QTL direction are derived from the previously mentioned data set (see *QTL sign test*). One confounding factor in this analysis was that studies conducted on small mapping populations or, analogously, using a small number of markers, can overestimate QTL effect size [30,31]. The number of QTL detected, a rough indicator of the mapping power of the study, was significantly higher for traits under biotic selection (mean of 3.9) than abiotic selection (mean of 3.5), suggesting that small-effect QTL are less likely to be detected for abiotic traits (Mann-Whitney U test, p = 0.0218). Our data show the opposite of this predicted trend (see below), suggesting any differences in mapping power did not bias our results.

**Figure 1:**
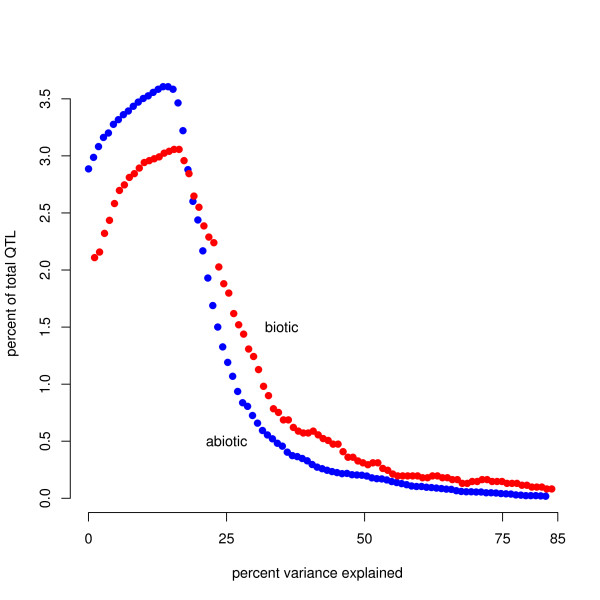
**Probability distribution derived via applying a moving average to the data of QTL effect size (PVE) of abiotic and biotic traits (see *methods*)**. The figure includes data from all accessions (N = 229 for biotic, 1721 for abiotic).

**Figure 2:**
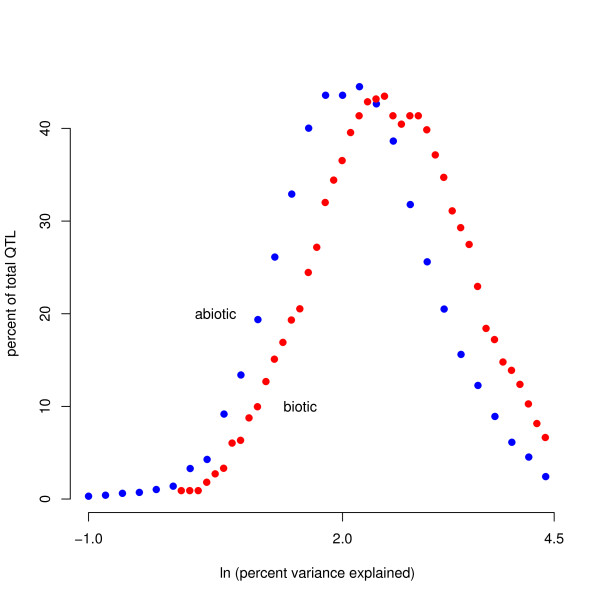
**Log-transformed abiotic and biotic calculated PDFs (see *methods*)**. Sample sizes are the same as Figure 1.

All PVE values were log-transformed before statistical analyses to conform to assumptions of normality (Figure 1 shows untransformed data; N = 229 for biotic, 1721 for abiotic). The transformed biotic data were normally distributed (Shapiro-Wilk W statistic = 0.9938, p = 0.4656) but the abiotic data were not (Shapiro-Wilk W statistic = 0.9664, p < 2.2e-16). In order to show the frequency distribution of QTL of different sizes, we calculated a probability density function (PDF) for both data sets, for both untransformed data and transformed data (Figures 1 and 2, respectively), by using a moving average method. This method generates a PDF by sequentially tabulating the number of observations falling within a set bin size across a nearly continuous series of values of the predictor variable [32].

Though the transformed biotic data set conformed to the assumptions of normality, due to violation of the assumptions of normality in the transformed abiotic data set, we performed both a Kruskal-Wallis test and a Student's t-test to determine if the distributions were different. Due to a difference in sample sizes between abiotic and biotic data sets, we also conducted nonparametric bootstrapping to assess differences in QTL effect sizes. We bootstrapped a sample of the same size as the biotic data set from the abiotic data set, and calculated a mean and variance of these bootstrapped replicates. We also bootstrapped samples from a combined data set including data from biotic and abiotic traits, and calculated a mean and variance from this second set of bootstrapped data. Finally, to test for the influence of confounding variables, we conducted AIC tests on a series of nested GLM models that used abiotic v. biotic status, cross type, natural v. laboratory status, and mating system as predictors of QTL effect sizes.

## Results

For both sets of analyses, there were far fewer traits studied in natural accessions compared to laboratory accessions. For the sign test, 103 out of 662 traits were studied in natural systems, and for effect size, 286 out of 1950 QTL were detected in natural accessions (see [Additional file 1] and [Additional file 2]; see [Additional file 3] for references). For both data sets (sign test and PVE), we conducted statistical analyses on a combined data set that included both natural and laboratory accessions. A chi-square test indicates that biotic traits are over-represented in the natural data set, but under-represented in the laboratory data set (p < 2.2e-16 for effect size and p = 0.004382 for effect direction). If researchers are selecting for extreme phenotypes in traits under abiotic selection, for subsequent use as parents of recombinant inbred lines (RILS) and QTL mapping analyses, one might predict fewer antagonistic QTL or larger PVE in these abiotic traits due to stronger selection. However, this predicted trend runs counter to the observed pattern (see below), supporting our inclusion of both natural and laboratory data sets in our analyses.

For the sign test, the majority of traits were classified as under abiotic selection (588 out of 662). Similarly, in the effect size data set, the majority of QTL detected (1721 out of 1950) controlled traits under abiotic selection. This analysis included organisms from 10 families, with the vast majority of traits mapped in Brassicaceae, 87.7% for QTL sign test and 89.3% for effect size (almost entirely *Arabidopsis thaliana*).

### Fractions of antagonistic QTL

Abiotic traits had, on average, 3.76 QTL per trait and biotic traits had 5.31 QTL per trait (for natural only, 3.70 and 4.82, respectively). Since a sign test requires at least 6 or more detected QTL per trait, our analysis of individual traits' deviations from neutrality was limited to 99 traits under abiotic selection and 23 traits under biotic selection, indicated in [Additional file 1] by the presence of a p-value. In all accessions, 5 of 23 of the tested biotic traits deviated significantly from neutrality, compared to 5 of 99 of tested abiotic traits. Fisher's exact test indicated that these proportions were significantly different from one another (p = 0.0353). The traits that deviated from neutrality are shown in Table 1.

In addition to testing for selection on individual traits, we also tested for directional selection on entire groups of traits. We summed all antagonistic and total QTLs detected for each trait across abiotic and biotic trait groupings, using data from all traits for which more than 1 QTL was detected, and tested these fractions for deviations from neutrality. As expected, fractions of antagonistic QTL in both types of traits significantly deviated from neutrality, indicating positive selection on both abiotic and biotic trait types (Table 2). Biotic traits had a lower fraction of antagonistic QTL, suggesting stronger directional selection on these types of traits [4], but a G test on the summed fraction, that included abiotic v. biotic status, cross type, and mating system as categorical variables, indicated that including abiotic v. biotic status and the associated interactions in the model did not improve its predictive power (p = 0.1732 for the abiotic v. biotic*fraction interaction term, 0.7312 for abiotic v. biotic*cross type*fraction, 0.1224 for abiotic v. biotic*mating system*fraction). Interestingly, previous work has shown that cross type, but not mating system, is an important predictor of fraction of antagonistic QTL in wild species [4]. In our model, including the mating system* fraction interaction improved the predictive power of the model (p < 0.0001), but the cross type*fraction interaction did not (p = 0.5461). The fraction of antagonistic QTL was significantly higher in crosses between selfers than in crosses between outcrossers (Mann-Whitney U-test, p = 0.002), in support of the findings of Rieseberg et al. [4] for wild species. For natural accessions only, we could not conduct this G test due to lack of power.

**Table 2:** Summed fractions of antagonistic QTL

	fraction of antagonistic QTL	antagonistic QTL	total QTL	p
abiotic	0.3079	649	2108	< 0.0001

biotic	0.2706	105	388	< 0.0001

Fraction of antagonistic QTL for all accessions, summing all QTL detected for all traits in each category. P values calculated using the normal approximation of the QTL sign test for when N is large [3].

We determined the effect of abiotic v. biotic status on the fraction of antagonistic QTL of individual traits, as well as accounted for the potentially confounding influence of auxiliary variables, by using AIC_c _criteria to select between nested GLM models. We used the fraction of antagonistic QTL controlling a given trait as the response variable, weighted by the total number of QTL [33], and the explanatory variables mating type, cross type, and abiotic v. biotic status. We included total QTL as a weighting factor because Reiseberg et al. [4] found total QTL to be significant in an ANOVA of fraction of antagonistic QTL. Removing abiotic v. biotic status from this full model, as well as any interactions including abiotic v. biotic status, marginally decreased the likelihood of the model, but AIC_c _criteria, which penalizes for an increase in parameter number, showed strong support for the reduced model that excluded abiotic v. biotic status (Table 3a). Number of QTL detected was significantly higher in biotic traits when analyses were confined to natural accessions (Mann-Whitney U test, p = 0.04), and AIC results were not different from those obtained with the full data set.

**Table 3:** AIC_c _comparisons of nested models for fraction of antagonistic QTL and QTL effect size

Model	number of parameters	LL	AIC_c_	AIC_c _weights
A: fraction of antagonistic QTL				

global	16	808.24	-1583.46	0.0005

abiotic/biotic removed	8	807.43	-1598.60	0.9994

B: QTL effect size				

global	16	-1597.42	3227.12	0.82

abiotic/biotic removed	8	-1607.06	3230.20	0.18

Nested GLM models of fraction of antagonistic QTL (A), and QTL effect size (B). LL (log likelihood) values and AIC_c _values, used to calculate AIC_c _weights, are shown for more complex global models including all parameters, as well as models with abiotic v. biotic status removed. AIC_c _weights indicate the relative support of the data for each of the models.

### QTL effect size

Most QTL were small-effect, with fewer QTL of larger effect, a distribution consistent with Orr's [10] model of adaptation, which predicts that the distribution of factors should follow roughly a negative exponential distribution (Figure 1). 83% of QTL detected were less than 20 PVE and 55% of QTL were less than 10 PVE (for natural accessions only, 60% and 31%, respectively). Small-effect QTL are only reliably detected when marker density is high and sample size is large [30,31]; therefore, because many studies did not have a high marker density or large sample size, the number of small-effect QTL is likely underestimated. However, because this underestimation is true for both abiotic and biotic traits, this limitation is unlikely to affect our comparison of these two trait types. Additionally, if studies of biotic traits detected fewer QTL on average, then our data would be more likely to overestimate effect sizes in traits under biotic selection. However, the mean number of QTL detected per trait was significantly higher in traits under biotic selection (Mann-Whitney U test, p = 0.02), suggesting that any bias caused by detection power would lead to an underestimation of QTL effect size in traits under biotic selection. The mean PVE was 12.103 for traits under abiotic selection and 18.140 for traits under biotic selection (Figures 1, 2) (for natural accessions only, the mean PVE for abiotic was 19.7, and for biotic was 21.4). We also found that QTL controlling flowering-related traits had a significantly higher PVE than did those controlling non-flowering related traits (mean of 20 and 14 PVE, respectively, Wilcoxon test, p = 0.0206).

We found a significant difference in the distribution of the effect sizes of traits under abiotic and biotic selection using a Kruskal-Wallis one-way analysis of variance (Kruskal-Wallis chi-squared = 53.3806, p = 2.748e-13). A Student's t-test yielded similar results (t = 7. 7191, p = 1.711e-13; Figure 1). We found identical results using a bootstrapping approach (data not shown). For natural accessions only, biotic traits had significantly greater PVE than abiotic traits according to a one tailed Student's t-test (p = 0.028), and the bootstrapping analysis supported this conclusion, but a Kruskal-Wallis test found no differences.

In order to control for any systematic differences in cross type or species characteristics, we fit a series of GLM models to test for these variables' effects on differences in QTL effect sizes in biotic and abiotic traits. We found that excluding abiotic v. biotic status and all the interactions including abiotic v. biotic status decreased the predictive power of the global model, according to both log likelihood and AIC_c _criteria (Table 3b). PVE was larger for biotic traits, outcrossers, natural accessions, and interspecific crosses. For natural accessions only, PVE was marginally higher in selfers than in outcrossers and higher in intraspecific crosses than in interspecific crosses.

We also conducted the same analyses for a subset of the data for effect size, including only data from traits for which 6 or more QTL were detected, in order to correct for any effect of reduced mapping density or sample size in traits under biotic selection that might overestimate QTL effect sizes. Results were quantitatively identical, with the exception of the GLM model fitting analysis. For this analysis, the log-likelihood values indicated the full model (that included abiotic v. biotic status) was a better fit to the data, but AIC_c _criteria, which penalizes for an increase in the number of parameters, supported the reduced model that did not include abiotic v. biotic status (Table 4). We did not perform this analysis on the natural accessions data set because so few traits met this criterion.

**Table 4:** AIC_c _comparisons of models for fraction of antagonistic QTL and QTL effect size (> 5 QTL)

Model	number of parameters	LL	AIC_c_	AIC weights
QTL effect size, global	15	-726.638	1485.90	0.0046

QTL effect size, reduced	8	-729.500	1475.16	0.9954

Nested GLM models of fraction of QTL effect size, including only QTL controlling traits for which more than 5 QTL were detected. LL (log likelihood) values and AIC_c _values, used to calculate AIC weights, are shown for more complex global models including all parameters, as well as models with abiotic v. biotic status removed. AIC weights indicate the relative support of the data for each of the models.

## Discussion

Both traits under abiotic and biotic selection were shown to be under directional selection using the QTL sign test. Though we found a lower proportion of antagonistic QTL in traits under biotic selection when compared to traits under abiotic selection, this difference was not robust to the confounding influences of cross type (interspecific v. intraspecific cross) or mating system (selfing v. outcrossing), as indicated by our G-test results. However, in our tests for directional selection on individual traits, confined to those traits for which 6 or more QTL were detected, we found a higher proportion of individual traits under biotic selection deviating from neutrality than traits under abiotic selection.

Effect size showed a much stronger pattern: traits under biotic selection had, on average, a larger PVE than traits under abiotic selection, with abiotic v. biotic status a strong predictor of effect size even when cross type and mating system were considered. The small difference in means between the two trait groups is likely due to the diluting influence of QTL of small effect detected across all trait types. Our results suggest that QTL of large effect are more important in determining an organism's phenotypic response to its biotic environment than in mediating adaptations to its abiotic environment.

### Fraction of antagonistic QTL

Our results confirmed Rieseberg et al.'s [4] findings showing that directional selection is an important mechanism of phenotypic differentiation across a wide range of plant species and traits. Previous studies have assumed that lower fractions of antagonistic QTL indicate stronger selective pressure during the adaptive walk during which that trait was driven to fixation [3,4]. Rieseberg et al. [4] found differences in the fraction of antagonistic QTL across types of traits, and used these differences to infer relative strengths of selective pressure on morphological, life history, and timing traits. Rieseberg et al. [4] found a higher fraction of antagonistic QTL in intraspecific v. interspecific crosses, morphological traits compared to life history traits, and developmental events when compared to all other traits, but did not compare the fraction of antagonistic QTL in traits under abiotic v. biotic selection.

We did not detect a higher fraction of antagonistic QTL in traits under biotic selection, as would be predicted if the adaptive landscape were more temporally variable than for traits under abiotic selection. However, these categories were confounded with other variables. In our study, more biotic traits (29% of total number of biotic traits) were mapped in interspecific crosses than abiotic traits (3% of total number of abiotic traits). This bias may have prevented us from detecting differences in fractions of antagonistic QTL between abiotic and biotic traits independent of cross type, because, in previous work, interspecific crosses have lower fractions of antagonistic QTL than intraspecific crosses in wild populations [4]. Low fractions of antagonistic QTL in interspecific crosses suggest that phenotypic differences between species are likely due to consistent directional selection, whereas consistent directional selection is less common in intraspecific differentiation [4]. In wild populations, mating system, or selfing v. outcrossing mode of reproduction, has not been shown to influence the proportion of antagonistic QTL [4], but, in our data set, the strong association of selfing with intraspecific crosses, due to the large number of *Arabidopsis thaliana *crosses, likely contributed to mating system being a significant predictor of fraction of antagonistic QTL. We were also unable to disentangle the effect of cross type and mating system from abiotic v. biotic status for individual traits (Table 1). We found significant deviations from neutrality under both herbivore- and pollinator-mediated selective pressures, suggesting that directional selection is not confined to one type of biotic selective pressure.

Lower fractions of antagonistic QTL can indicate more consistent (i.e., less variable) selective pressure [4]. We detected no differences in fraction of antagonistic QTL across trait types. The strength, direction, and form of selection are known to vary considerably [13,34], and, further, vary systematically with trait type and selection medium. Prior work has found stronger selection on morphological traits as compared to life history or phenology traits, as well as stronger sexual and fecundity selection compared to selection for survival [34]. However, we found no evidence for differences in the strength of selection pressure between traits under abiotic and biotic selection. In order to test if traits under abiotic v. biotic selection differ in variability, we used data amassed in a recent review of temporal variation in selection pressures [13], reclassifying the traits identified in this study as under abiotic or biotic selection. These two categories did not differ in almost all metrics of variability in strength or direction. This analysis suggests that though variability in selection exists, traits under abiotic and biotic selection experience similar variability in selective pressure, leading to a lack of significant differences in antagonistic QTL fractions in our analysis.

An alternative mechanistic explanation for our finding of no differences in the fraction of antagonistic QTL is that antagonistic QTL may arise due to pleiotropic effects of selection on other traits. Pleiotropic effects have been found for a wide variety of traits [6,35-37] and are known to result in high fractions of antagonistic QTL [38]. Therefore, nonsignificant differences in the fraction of antagonistic QTL may have arisen because QTL under abiotic and biotic selection experience similar levels of pleiotropy. Finally, another reason we may not have found differences in fractions of antagonistic QTL between traits subject to abiotic and biotic selective pressures is that abiotic selection can also have discrete optima of high fitness in their adaptive landscapes; for example, selection has resulted in discrete strategies for adaptation to drought and freezing, such as avoidance or tolerance. Similarly, discrete habitat types, such as serpentine v. non-serpentine soils, can select for one of a series of alternate genotypes. Here we assume that discrete adaptive syndromes with deep valleys between peaks are more common in traits under biotic selection, but this may not be the case. It is possible that QTL studies mapping abiotic adaptations to discrete syndromes may have obscured any signal of differences between fractions of antagonistic QTL in traits under abiotic v. biotic selection.

### QTL effect size

Effect size distributions were consistent with a variety of previous work supporting the geometric model of QTL effect size distributions (reviewed in ref. [8]). Under the infinitesimal model, a large number of small-effect QTL operate to control traits [7], but we found that many QTL are of large effect. A QTL that explains more than 20% of the variance in phenotype is generally considered to be of large effect [39]. We found that for 10.7% of the traits in this analysis at least one QTL fitting this criterion was detected, and for 2% of traits in this analysis at least one QTL that explained more than 50% of the variance was detected. These data suggest that QTL of large effect, and even very large effect, are fairly common, in contrast to the predictions of the infinitesimal model. Our data further suggest that the simplifying assumption of the use of the infinitesimal model in quantitative genetic theory may be unrealistic.

Effect sizes differed between traits under abiotic and biotic selection, putatively due to differences in the adaptive landscapes associated with these traits. These differences in the adaptive landscape can be described by Maynard Smith's model of mutational space [5,8,40-42], which assumes that wild type populations are highly fit and close to the phenotypic optimum; the predicted distribution of QTL effect sizes resulting from this scenario is similar to Fisher's geometric model [5,38]. The assumption that populations are highly fit is accurate for traits under selective pressure by abiotic forces, but may not be accurate for traits under selective pressure by biotic forces; while populations are likely well-adapted to their abiotic environment, and track changes in temperature and precipitation relatively closely, biotic selective forces can change suddenly, because they are controlled by the distributions of other organisms. Thus, for traits under biotic selection, the adaptive landscape should be more temporally variable [43,18], which should consistently move formerly highly fit populations far away from an adaptive peak. Though current models provide no general prediction about the effect of shifting phenotypic optima on QTL effect size [5], QTL fixed early in an adaptive walk, or when populations are far from their phenotypic optimum, are larger than QTL fixed later [9,44]. Therefore, populations that repeatedly move far away from a phenotypic optimum and then need to begin an adaptive walk again may be more likely to accumulate relatively large-effect QTLs.

In addition to temporal variability, many traits under biotic selection in our study are characterized by a landscape that harbors many adaptive peaks, with deep valleys between the peaks associated with discrete adaptive syndromes. A meta-analysis on selection gradients [34] suggests that disruptive selection might be as common in nature as stabilizing selection, suggesting that many adaptive landscapes may harbor multiple adaptive peaks. Populations located within striking distance of a number of adaptive peaks may make large adaptive steps towards any one of those peaks [9,45]. For example, pollination syndromes are alternative adaptive peaks that may often likely have deep valleys of low fitness between them [46]. Such an adaptive landscape might favor the fixation of large mutations in order to move sufficiently close to an alternative adaptive peak to experience a fitness benefit. Similarly, the adaptive landscape for the production of highly specialized herbivore- specific defensive compounds likely have very deep valleys between adaptive peaks. Our finding of no difference in fractions of antagonistic QTL for traits under biotic v. abiotic selection suggests that, rather than large-effect QTL resulting from high temporal or spatial variability in biotic selective pressures, which should be accompanied by high fractions of antagonistic QTL in traits under biotic selection, large-effect QTL may be favored in traits under biotic selection due to the presence of discrete adaptive syndromes and deep valleys of low fitness in the adaptive landscape.

Supporting these hypotheses about the adaptive landscape of biotic interactions, drastic differences in pollination syndromes [47,48] and herbivore defenses [49,50] have been observed in closely related organisms. We may expect a greater number of large-effect QTL controlling these traits. In fact, we would predict large effect QTL whenever the depth of valleys between adaptive peaks is similar to that experienced by traits under biotic selection. In particular, flowering-related traits had large PVE values, and may be driving the observed differences in QTL effect size between traits under abiotic and biotic selection. Similarly, traits involved in crop domestication, subject to sudden selective pressures by humans, and with strongly decreased fitness in intermediate phenotypes, are almost universally controlled by QTL of large effect (with the exception of sunflower; see ref. [51]). The most well-known example is maize, where only five genomic regions control most of the phenotypic differences between domesticated maize and its wild progenitor teosinte [52,53]. This scenario also suggests that populations living in areas of high species richness, such as the tropics, should have increased average QTL effect size [18] due to an increase in the number of phenotypic optima and an increase in the dimensions of niche space [18,54,55].

## Conclusions

Our analysis suggests interesting differences in the adaptive landscape for traits under abiotic and biotic selection. Though the geometric model of adaptation predicts that high temporal or spatial variability in selective pressure by biotic forces should favor fixation of large-effect QTL, our finding of no differences in the fractions of antagonistic QTL for traits under biotic v. abiotic selection suggests that the large-effect QTL detected in this study cannot be attributed to a shifting adaptive landscape. On the contrary, our results suggest a novel mechanism by which large-effect QTL may be favored: differences in effect size, coupled with a lack of differences in the fraction of antagonistic QTL, suggest that the presence of discrete adaptive syndromes in traits under biotic selection may favor the fixation of large- effect QTL, independent of increased temporal or spatial variability in biotic selective pressures.

Though this study highlights the potential of QTL studies for evaluating hypotheses about the genetic architecture of traits, it also showcases the limitations of available data. Specifically, most QTL mapping is confined to only a few laboratory-based ecotypes and limits conclusions about the genetic architecture of traits in natural populations. In addition, most QTL mapping studies are conducted on traits under abiotic selection, with a paucity of traits under biotic selection. Furthermore, most of the traits under biotic selection for which QTL are mapped are floral display traits, which our study shows have larger-effect QTL than other traits under abiotic selection, rather than traits conferring resistance to naturally occurring herbivores or pathogens. Researchers conducting QTL mapping studies of pollination syndromes, which represent the majority of traits under biotic selection identified in this study, may choose to study discrete syndromes; thus, our results may be more reflective of adaptation to pollinators rather than biotic factors per se. A stronger emphasis on mapping QTL in traits controlled by biotic selection, and specifically on disease or herbivory resistance in natural populations, would facilitate comparisons of the genetic architecture of traits under biotic and abiotic selection. Our analysis also displays the difficulty of comparing QTL architecture across studies, due to a large number of factors that influence statistical power, including the number and distribution of markers, crossing design, and number of recombinants. QTL studies are becoming increasingly feasible and less expensive, with the potential to map a wide variety of traits in a variety of organisms. With the advent of more affordable QTL mapping techniques, we anticipate an increase in the data available to perform such analyses.

## Abbreviations

QTL: indicates quantitative trait loci; PVE: indicates percent variance explained.

## Authors' contributions

AL assembled data, conducted statistical analyses, and drafted the manuscript. KK conceived of the study and helped draft the manuscript. Both authors read and approved the final manuscript.

## Supplementary Material

Additional file 1 **Antagonistic QTL fractions**. Contains data on trait classifications, organism used, total QTL, antagonistic QTL fractions, individual traits' deviations from neutrality, as well as references for the study from which the data was taken, for all traits used in the sign test analyses presented in this paper. References contained in supplemental word document [Additional file 3]. Click here for file

Additional file 2 **PVE values**. Contains data on PVE (percent variance explained), trait classifications, and organism used, for all traits used in the PVE analyses presented in this paper. References contained in supplemental word document [Additional file 3]. Click here for file

Additional file 3 **Contains numbered references for studies included in Additional files **1**and **2. Click here for file
