# Supplementary material for: Comparing the adaptive landscape across trait types: larger QTL effect size in traits under biotic selection
Source: BMC Evol Biol. 2011 Mar 7;11:60. doi: 10.1186/1471-2148-11-60 (PMC3061918; doi:10.1186/1471-2148-11-60)
Supplement: Additional file 3 — Contains numbered references for studies included in Additional files 1and 2. [file 1471-2148-11-60-S3.DOC]

**References**

1. Kowalski SP, Lan TH, Feldman KA, Paterson AH: **QTL mapping of naturally-occurring variation in flowering time of *Arabidopsis thaliana****. Mol Gen Genet* 1994, **245:** 548-555.

2. Bradshaw HD, Stettler RF: **Molecular-genetics of growth and development in Populus 4. Mapping QTLs with large effects on growth, form and phenology traits in a forest tree.** *Genetics* 1995, **139:** 963-973.

3. Clarke JH, Mithen R, Brown JKM, Dean C: **QTL analysis of flowering time in *Arabidopsis thaliana***. *Mol Gen Genet* 1995, **248:** 278-286.

4. Hombergen EJ, Backmann K: RPAD mapping of 3 QTLs determining trichome formation in Microseris hybrid H27 (Asteraceae, Lactuceae). *Theor Appl Genet* 1995, **90:** 853-858.

5. Kuittinen H, Sillanpaa MJ, Savolainen O: **Genetic basis of adaptation: flowering time in *Arabidopsis thaliana***. *Theor Appl Genet* 1997, **95:** 573-583.

6. Van Der Schaar W, Alonso-Blanco C, Leon-Kloosterziel KM, Jansen RC, Van Ooijen, JW, Koornneef, M: **QTL analysis of seed dormancy in Arabidopsis using recombinant inbred lines and MQM mapping**. *Heredity* 1997*,* 79**:**190-200.

7. Alonso-Blanco C, El-Assal SED, Coupland G, Koornneef M: **Analysis of natural allelic variation at flowering time loci in the Landsberg erecta and Cape Verde islands ecotypes of *Arabidopsis thaliana***. *Genetics* 1998, **149:** 749-764.

8. Bradshaw HD, Otto KG, Frewen BE, McKay JK, Schemske DW: **Quantitative trait loci affecting differences in floral morphology between two species of monkeyflower (Mimulus)**. *Genetics* 1998, 149**:** 367-382.

9. Mitchell-Olds T, Pedersen D. **The molecular basis of quantitative genetic variation in central and secondary metabolism in Arabidopsis**. *Genetics* 1998*,* **149**: 739-747.

10. Stratton DA: **Reaction norm functions and QTL-environment interactions for flowering time in Arabidopsis thaliana**. *Heredity* 1998, **81:** 144-155.

11. Alonso-Blanco C, Blankestijn-de Vries H, Hanhart CJ, Koornneef M: **Natural allelic variation at seed size loci in relation to other life history traits of *Arabidopsis thaliana*.** *Proc Natl Acad Sci U S A* 1999, **96**: 4710-4717.

12. Swarup K, Alonso-Blanco C, Lynn JR, Micheals SD, Amasino RM, Koornneef M, Millar AJ: **Natural allelic variation identifies new genes in the Arabidopsis circadian system.** *Plant Journal* 1999, **20**: 67-77.

13. Bentsink L, Alonso-Blanco C, Vreugdenhil D, Tesnier K, Groot SPC, Koornneef, M: **Genetic analysis of seed-soluble oligosaccharides in relation to seed storability of Arabidopsis**. *Plant Physiol* 2000, **124**: 1595-1604.

14. Frewen BE, Chen THH, Howe GT, Davis J, Rohde A, Boerjan W, Bradshaw HD: **Quantitative trait loci and candidate gene mapping of bud set and bud flush in Populus.** *Genetics* 2000, 154**:** 837-845.

15. Hurme P, Sillanpaa MJ, Arjas E, Repo T, Savolainen O: **Genetic basis of climatic adaptation in Scots pine by Bayesian quantitative trait locus analysis**. *Genetics 2000,* 156**:** 1309-1322.

16. Juenger T, Purugganan M, Mackay TFC: **Quantitative trait loci for floral morphology in *Arabidopsis thaliana***. *Genetics* 2000,156**:** 1379-1392.

17. Ungerer MC: **Role of gene interactions in evolution**. *PhD thesis*. Indiana University, Dept. of Biology; 2000.

18. Whitkus R, Doan H, Lowrey TK: **Genetics of adaptive radiation in Hawaiian species of Tetramolopium (Asteraceae). III. Evolutionary genetics of sex expression**. *Heredity* 2000, **85**: 37-42.

19. Moritz,M, Kadereit K: **The genetics of evolutionary change in *Senecio vulgaris* L.: A QTL mapping approach.** *Plant Biology* 2001, **3**: 544-552.

20. Lambrix V, Reichelt M, Mitchell-Olds T, Kliebenstein DJ, Gershenzon J: **The Arabidopsis epithiospecifier protein promotes the hydrolysis of glucosinolates to nitriles and influences *Trichoplusia ni* herbivory**. *Plant Cell Online* 2001, **13**: 2793-2807.

21. Linde M, Diel S, Neuffer B: **Flowering ecotypes of *Capsella bursa-pastoris* (L.) Medik. (Brassicaceae) analysed by a cosegregation of phenotypic characters (QTL) and molecular markers.** *Ann Bot* 2001, **87**: 91-99.

22. Pepper P, Corbett C, Kang K: **Natural variation in Arabidopsis seedling photomorphogenesis reveals a likely role for TED1 in phytochrome signalling**. *Plant Cell Environ* 2002, 25**:** 591-600.

23. Borevitz JO, Maloof JN, Lutes J, Dabi T, Redfern JL, Trainer GT, Werner JD, Asami I, Berry CC, Weigel D, Chory J: **Quantitative trait loci controlling light and hormone response in two accessions of *Arabidopsis thaliana*.** *Genetics* 2002, 160**:** 683-696.

24. Kliebenstein D, Pedersen D, Barker B, Mitchell-Olds T: **Comparative Analysis of Quantitative Trait Loci Controlling Glucosinolates**, **Myrosinase and Insect Resistance in *Arabidopsis thaliana***. *Genetics* 2002, **161**: 325-332.

25. Perez-Perez JM, Serrano-Cartagena J, Micol, JM: **Genetic Analysis of Natural Variations in the Architecture of *Arabidopsis thaliana*** **vegetative leaves.** *Genetics* 2002, **162:** 893-915.

26. Ungerer MC, Halldorsdottir SS, Modliszewski JL, Mackay TF, Purugganan MD: **Quantitative trait loci for inflorescence development in *Arabidopsis thaliana***. *Genetics* 2002, **160**:1133-1151.

27. Quesada Q, Garcia-Martinez G-M, Piqueras P, Ponce P, Micol M: **Genetic architecture of NaCl tolerance in Arabidopsis**. *Plant Physiol* 2002, 130**:** 951-963.

28. Weinig C, Ungerer MC, Dorn LA, Kane NC, Toyonaga Y, Halldorsdottir SS, Mackay TFC, Purugganan MD, Schmitt J: **Novel loci control variation in reproductive timing in Arabidopsis thaliana in natural environments.** *Genetics* 2002 **162**:1875-1884.

29. Botto JF, Alonso-Blanco C, Garzaron I, Sanchez RA, Casal JJ: **The Cape Verde Islands allele of cryptochrome 2 enhances cotyledon unfolding in the absence of blue light in Arabidopsis**. *Plant Physiol* 2003, **133:** 1547-1556.

30. Hoekenga OA, Vision TJ, Shaff JE, Monforte AJ, Lee GP, Howell SH, Kochian LV: **Identification and characterization of aluminum tolerance loci in Arabidopsis (Landsberg erecta x Columbia) by quantitative trait locus mapping. A physiologically simple but genetically complex trait.** *Plant Physiol* 2003, **132**: 936-948.

31. Godiard G, Sauviac S, Torii T, Grenon G, Mangin B, Grimsley NH, Marco Y: **ERECTA, an LRR receptor-like kinase protein controlling development pleiotropically affects resistance to bacterial wilt**. *Plant Journal* 2003, **36:** 353-365.

32. Loudet O, Chaillou C, Krapp K, Daniel-Vedele DV: **Quantitative trait loci analysis of water and anion contents in interaction with nitrogen availability in *Arabidopsis thaliana***. *Genetics* 2003, **163**: 711-722.

33. Ungerer M, Halldorsdottir SS, Purugganan MD, Mackay TFC: **Genotype-environment interactions at quantitative trait loci affecting inflorescence development in *Arabidopsis thaliana***. *Genetics* 2003, **165**: 353-365.

34. Loudet L, Chaillou C, Merigout M, Talbotec T, Daniel-Vedele DV: **Quantitative trait loci analysis of nitrogen use efficiency in Arabidopsis**. *Plant Physiol* 2003, **131**: 345-358.

35. Weinig C, Dorn LA, Kane NC, German ZM, Halldorsdottir SS, Ungerer MC, Toyonaga Y, Mackay TFC, Purugganan MC, Schmitt J*.* **Heterogeneous selection at specific loci in natural environments in *Arabidopsis thaliana***. *Genetics*  2003, **165:** 321-329.

36. Weinig C, Stinchcombe JR, Schmitt J: **QTL architecture of resistance and tolerance traits in *Arabidopsis thaliana* in natural environments**. *Mol Ecol* 2003, **12:** 1153-1163.

37. Clerkx EJM, El-Lithy ME, Vierling E, Ruys GJ, Blankestijin-De Vries H, Groot SPC, Vreugdenhil D, Koornneef M*:* **Analysis of natural allelic variation of Arabidopsis seed germination and seed longevity traits between the accessions Landsberg erecta and Shakdara, using a new recombinant inbred line population**. *Plant Physiol* 2004, **135:** 432-443.

38. Denby KJ, Kumar P, Kliebenstein DJ: **Identification of *Botrytis cinerea* susceptibility loci in *Arabidopsis thaliana***. *Plant Journal* 2004, **38**: 473-486.

39. Harada H, Kuromori K, Hirayama H, Shinozaki S, Leigh L: **Quantitative trait loci analysis of nitrate storage in Arabidopsis leading to an investigation of the contribution of the anion channel gene, AtCLC-c, to variation in nitrate levels**. *J Exp Bot* 2004, **55**: 2005-2014.

40. Hobbs DH, Flintham JE, Hills MJ: **Genetic control of storage oil synthesis in seeds of Arabidopsis**. *Plant Physiol* 2004, **136:** 3341-3349.

41. Payne KA, Bowen HC, Hammond JP, Hampton CR, Lynn JR, Mead A, Swarup K, Bennet MJ, White PJ, Broadley MR: **Natural genetic variation in caesium (Cs) accumulation by *Arabidopsis thaliana***. *New Phytol* 2004, **162**: 535-548.

42. El-Lithy M, Clerkx EJM, Ruys GJ, Koorneef M, Vreugdenhill D. Quantitative trait locus analysis of growth-related traits in a new Arabidopsis recombinant. *Plant Physiol* 2004, **135:** 444-458.

43. Lall S, Nettleton D, DeCook R, Che P, Howell SH: **Quantitative trait loci associated with adventitious shoot formation in tissue culture and the program of shoot development in arabidopsis**. *Genetics* 2004, **167**: 1883-1892.

44. Sergeeva LI, Vonk J, Keurentjes JJB, van der Plas, LHW, Koornneef M, Vreugdenhill D: **Histochemical analysis reveals organ-specific quantitative trait loci for enzyme activities in Arabidopsis**. *Plant Physiol* 2004, **134**: 237-245.

45. Verhoeven KJF, Vanhala TK, Biere A, NevoE, van Damme JMM: **The genetic basis of adaptive population differentiation: a quantitative trait locus analysis of fitness traits in two wild barley populations from contrasting habitats**. *Evolution*  2004, **58**: 270-283.

46. Alonso-Blanco C, Gomez-Mena C, Llorente F, Koornneef M, Salinas J, Martinez-Zapater JM: **Genetic and molecular analyses of natural variation indicate CBF2 as a candidate gene for underlying a freezing tolerance quantitative trait locus in Arabidopsis**. *Plant Physiol* 2005, **139**:1304-1312.

47. Edwards KD, Lynn JR, Gyula P, Nagy F, Millar AJ: **Natural allelic variation in the temperature-compensation mechanisms of the *Arabidopsis thaliana* circadian clock**. *Genetics* 2005, **170**: 387-400.

48. Llorente F, Alonso-Blanco C, Sanchez-Rodriguez C, Jordo L, Molina A: **ERECTA receptor-like kinase and heterotrimeric G protein from Arabidopsis are required for resistance to the necrotrophic fungus *Plectosphaerella cucumerina***. *Plant Journal*  2005, **43**: 165-180.

49. Hausmann NJ, Juenger TE, Sen S, Stowe KA, Dawson TE, Simms EL: **Quantitative trait loci affecting delta C-13 and response to differential water availability in *Arabidopsis thaliana***. *Evolution* 2005, **59**: 81-96.

50. Juenger T, Perez-Perez JM, Bernal S, Micol JL: **Quantitative trait loci mapping of floral and leaf morphology traits in *Arabidopsis thaliana*: evidence for modular genetic architecture**. *Evol Dev* 2005, **7**: 259-271.

51. Juenger TE, Sen S, Stowe KA, Simms EL: **Epistasis and genotype-environment interaction for quantitative trait loci affecting flowering time in *Arabidopsis thaliana***. *Genetica* 2005, **123**: 87-105.

52. Kobayashi Y, Furuta Y, Ohno T, Hara T, Koyama H: **Quantitative trait loci controlling aluminium tolerance in two accessions of *Arabidopsis thaliana* (Landsberg erecta) and Cape Verde Islands)**. *Plant Cell Environ* 2005, **28**:1516-1524.

53. Malmberg RL, Held S, Waits A, Mauricio R: **Epistasis for fitness-related quantitative traits in Arabidopsis thaliana grown in the field and in the greenhouse**. *Genetics* 2005, **171**: 2013-2027.

54. Martin NH, Bouck AC, Arnold ML: **Loci affecting long-term hybrid survivorship in Louisiana Irises: implications for reproductive isolation and introgression**. *Evolution* 2005, **59**: 2116-2124.

55. Mauricio R: **Ontogenetics of QTL: the genetic architecture of trichome density over time in *Arabidopsis thaliana***. *Genetica* 2005, **123**: 75-85.

56. Kover, PX, Wolf JB, Kunkel BN, Cheverud JM: **Genetic architecture of *Arabidopsis thaliana* response to infection by *Pseudomonas syringae***. *Heredity* 2005, **94**: 507-517.

57. Symonds VV, Godoy AV, Alconada T, Botto JF, Juenger TE, Casal JJ, Lloyd AM*:* **Mapping quantitative trait loci in multiple populations of *Arabidopsis thaliana* identifies natural allelic variation for trichome density**. *Genetics* 2005, **169**:1649-1658.

58. Juenger TE, McKay JK, Hausmann N, Keurentjes JJB, Sen S, Stowe KA, Dawson TE, Simms EL, Richards JH: **Identification and characterization of QTL underlying whole-plant physiology in *Arabidopsis thaliana*: delta C-13, stomatal conductance and transpiration efficiency**. *Plant Cell Environ* 2005, **28**: 697-708.

59. Barriere Y, Laperche A, Barrot L, Aurel G, Briand M, Jouanin L: **QTL analysis of lignification and cell wall digestibility in the Bay-0 x Shahdara RIL progeny of *Arabidopsis thaliana* as a model system for forage plant**. *Plant Sci* 2005, **168**: 1235-1245.

60. Assuncao AGL, Pieper B, Vromans J, Lindhout P, Aarts MGM, Schat H. **Construction of a genetic linkage map of *Thlaspi caerulescens* and quantitative trait loci analysis of zinc accumulation**. *New Phytol* 2006, **170**: 21-32.

61. Deniau AX, Pieper B, Ten Bookum WB, Linhout P, Aarts MGM, Schat H: **QTL analysis of cadmium and zinc accumulation in the heavy metal hyperaccumulator *Thlaspi caerulescens****. Theor Appl Genet* 2006, **113**: 907-920.

62. Bratteler M, Baltisberger M, Widmer A: **QTL analysis of intraspecific differences between two Silene vulgatis ecotypes**. *Ann Bot* 2006, **98**: 411-419.

63. Darrah C, Taylor BL, Edwards KD, Brown PE, Hall A, McWatters HG: **Analysis of phase of LUCIFERASE expression reveals novel circadian quantitative trait loci in Arabidopsis**. *Plant Physiol* 2006, **140**: 1464-1474.

64. El-Lithy, ME, Bentsink L, Hanhart CJ, Ruys GJ, Rovito DI, Broekhof JLM, van der Poel HJA, van Eijk MJT, Vreugdenhil D, Koornneef M: **New Arabidopsis recombinant inbred line populations genotyped using SNPWave and their use for mapping flowering-time quantitative trait loci.** *Genetics* 2006, **172**: 1867-1876.

65. Calenge F, Saliba-Colombani V, Mahieu S, Loudet O, Daniel-Vedele F, Krapp A: **Natural variation for carbohydrate content in Arabidopsis. Interaction with complex traits dissected by quantitative genetics**. *Plant Physiol* 2006, **141**: 1630-1643.

66. Fitz Gerald JN, Lehti-Shiu MD, Ingram PA, Deak KI, Biesiada T, Malamy JE: **Identification of quantitative trait loci that regulate Arabidopsis root system size and plasticity**. *Genetics* 2006, **172**: 485-498.

67. Galliot C, Hoballah ME, Kuhlemeier C, Stuurman J: **Genetics of flower size and nectar volume in Petunia pollination syndromes**. *Planta* 2006, **225**: 203-212.

68. Harada H, Leigh, R: **Genetic mapping of natural variation in potassium concentrations in shoots of *Arabidopsis thaliana***. *J Exp Bot*  2006, **57**: 953-960.

69. Sergeeva LI, Keurentjes JJB, Bentsink L, Vonk J, van der Plas LHW, Koornneef M, Vreugdenhill D: **Vacuolar invertase regulates elongation of *Arabidopsis thaliana* roots as revealed by QTL and mutant analysis**. *Proc Natl Acad Sci U S A* 2006, **103**: 2994-2999.

70. Bratteler M, Lexer C, Widmer A: **Genetic architecture of traits associated with serpentine adaptation of *Silene vulgaris***. *J Evol Biol* 2006, **19**: 1149-1156.

71. Mouille G, Witucka-Wall H, Bruyant MP, Loudet O, Pelletier S, Rihouey C, Lerouxel O, Lerouge P, Höfte H, Pauly M: **Quantitative trait loci analysis of primary cell wall composition in Arabidopsis**. *Plant Physiol*  2006, **141**:1035-1044.

72. Martin NH, Bouck AC, Arnold ML: **Detecting adaptive trait introgression between *Iris fulva* and *I. brevicaulis* in highly selective field conditions**. *Genetics* 2006, **172**: 2481-2489.

73. Reymond M, Svistoonoff S, Loudet O, Nussaume L, Desnos T: **Identification of QTL controlling root growth response to phosphate starvation in *Arabidopsis thaliana***. *Plant Cell Environ* 2006 **29**: 115-125.

74. Diaz C, Saliba-Colombani V, Loudet O, Belluomo P, Moreau L, Daniel-Vedele F, Morot-Gaudry J, Masciaux-Daubresse C: **Leaf yellowing and anthocyanin accumulation are two genetically independent strategies in response to nitrogen limitation in *Arabidopsis thaliana***. *Plant Cell Physiol* 2006, **47**: 74-83.

75. Luquez VMC, Sasal Y, Medrano M, Martin MI, Mijica M, Guiamet JJ: **Quantitative trait loci analysis of leaf and plant longevity in *Arabidopsis thaliana***. *J Exp Bot* 2006, **57**:1363-1372.

76. Zhang LH, Byrne PF, Pilon-Smits EAH: **Mapping quantitative trait loci associated with selenate tolerance in *Arabidopsis thaliana***. *New Phytol* 2006, **170**: 33-42.

77. Wentzell AM, Rowe HC, Hansen BG, Ticconi H, Halkier BA, Kliebenstein DJ: **Linking metabolic QTLs with network and cis-eQTLs controlling biosynthetic pathways**. *PLOS Genetics* 2007, **3**: 1687-1701.

78. Kusterer B, Piepho H, Utz HF, Schon CC, Muminovic J, Meyer RC, Altmann T, Melchinger AE: **Heterosis for biomass-related traits in Arabidopsis investigated by quantitative trait loci analysis of the triple testcross design with recombinant inbred lines**. *Genetics* 2007, **177**: 1839-1850.

79. Courbot M, Willems G , Motte P, Arvidsson S, Roosens N, Saumitou-Laprade P, Verbruggen N: **A major quantitative trait locus for cadmium tolerance in *Arabidopsis halleri* colocalizes with HMA4, a gene encoding a heavy metal ATPase**. *Plant Physiol* 2007, **144**:1052-1065.

80. Esch E, Szymaniak JM, Yates H, Pawlowski WP, Buckler ES: **Using crossover breakpoints in recombinant inbred lines to identify quantitative trait loci controlling the global recombination frequency**. *Genetics* 2007, **177**: 1851-1858.

81. Botto JF, Coluccio MP: **Seasonal and plant-density dependency for quantitative trait loci affecting flowering time in multiple populations of *Arabidopsis thaliana***. *Plant Cell Environ* 2007, **30**:1465-1479.

82. Keurentjes JJB, Bentsink L, Alonso-Blanco C, Harhart CJ, Blankestijn-De Vries H, Effgen S, Vreugdenhill D, Koornneef M: **Development of a near-isogenic line population of *Arabidopsis thaliana* and comparison of mapping power with a recombinant inbred line population**. *Genetics* 2007, **175**: 891-905.

83. Martin NH, Bouck AC, Arnold ML: **The genetic architecture of reproductive isolation in Louisiana irises: Flowering phenology**. *Genetics*  2007, **175**:1803-1812.

84. Mutic JJ, Wolf JB: **Indirect genetic effects from ecological interactions in *Arabidopsis thaliana***. *Mol Ecol* 2007, **16**: 2371-2381.

85. Kover PX, Cheverud J: **The genetic basis of quantitative variation in susceptibility of *Arabidopsis thaliana* to *Pseudomonas syringae* (Pst DC3000): evidence for a new genetic factor of large effect**. *New Phytol* 2007, **174**: 172-181.

86. Sulpice R, Tschoep H, Von Korff M, Bussis D, Usadel B, Hohne M, Witucka-Wall H, Altmann T, Stitt M, Gibon Y: **Description and applications of a rapid and sensitive non-radioactive microplate-based assay for maximum and initial activity of D-ribulose-1,5-bisphosphate carboxylase/oxygenase**. *Plant Cell Environ* 2007, **30**: 1163-1175.

87. Ikka T, Kobayashi Y, Iuchi S, Sakurai N, Shibata D, Kobayashi M, Koyama H: **Natural variation of *Arabidopsis thaliana* reveals that aluminum resistance and proton resistance are controlled by different genetic factors**. *Theor Appl Genet*  2007, **115**: 709-719.

88. Willems G, Drager DB, Courbot M, Gode C, Verbruggen N, Saumitou-Laprade P: **The genetic basis of zinc tolerance in the metallophyte *Arabidopsis halleri* ssp halleri (Brassicaceae): An analysis of quantitative trait loci**. *Genetics* 2007, **176**: 659-674.

89. Waters BM, Grusak MA: **Quantitative trait locus mapping for seed mineral concentrations in two *Arabidopsis thaliana* recombinant inbred populations**. *New Phytol* 2008, **179**: 1033-1047.

90. Zeng C, Han Y, Shi L, Peng L, Wang Y, Xu F, Meng J: **Genetic analysis of the physiological responses to low boron stress in *Arabidopsis thaliana***. *Plant Cell Environ* 2008, **31**: 112-122.

91. Rower HC, Kliebenstein DJ: **Complex Genetics Control Natural Variation in *Arabidopsis thaliana* Resistance to *Botrytis cinerea***. *Genetics* 2008,**180**: 2237-2250.

92. Lisec J, Meyer RC, Stenfath M, Redestig H, Becher M, Witucka-Wall H, Fiehn O, Torjek O, Selbig J, Altmann T, Willmitzer L: **Identification of metabolic and biomass QTL in *Arabidopsis thaliana* in a parallel analysis of RIL and IL populations**. *Plant Journal* 2008, **53**: 960-972.

93. Keurentjes JJB, Sulpice R, Gibon Y, Steinhauser M-C, Jingyuan F, Koornneef M, Stitt M, Vreugdenhill D: **Integrative analyses of genetic variation in enzyme activities of primary carbohydrate metabolism reveal distinct modes of regulation in *Arabidopsis thaliana***. *Genome Biol* 2008, **9**: R129.

94. Verhoeven KJF, Poorter H, Nevo E, Biere A: **Habitat-specific natural selection at a flowering-time QTL is a main driver of local adaptation in two wild barley populations**. *Mol Ecol* 2008, **17**: 3416-3424.

95. Laserna MP, Sanchez RA, Botto JF: **Light-related loci controlling seed germination in L*er* x Cvi and Bay-0 x Sha recombinant inbred-line populations of *Arabidopsis thaliana***. *Ann Bot* 2008, **102**: 631-642.

96. Loudet O, Michael TP, Burger BT, Le Mette C, Mockler TC, Weigel D, Chory J: **A zinc knuckle protein that negatively controls morning-specific growth in *Arabidopsis thaliana***. *Proc Natl Acad Sci U S A* 2008, **105**: 17193-17198.

97. Jubault M, Lariagon C, Simon M, Delourme R, Manzanares-Dauleux MJ: **Identification of quantitative trait loci controlling partial clubroot resistance in new mapping populations of *Arabidopsis thaliana***. *Theor Appl Genet* 2008, **117**: 191-202.

98. Martin, M., Sapir, S. & Arnold, A: **The genetic architecture of reproductive isolation in Louisiana Irises: Pollination syndromes and pollinator preferences**. *Evolution* 2008, **62**: 740-752.

99. McKay JK, Richards JL, Nemali KS, Sen S, Mitchell-Olds T, Boles S, Stahl E, Tierney W, Juenger TE: **Genetics of drought adaptation in *Arabidopsis thaliana* II. QTL analysis of a new mapping population, Kas-1 x Tsu-1**. *Evolution* 2008, **62**: 3014-3026.

100. O'Neill CM, Morgan C, Kirby J, Tschoep H, Deng PX, Brennan M, Rosas U, Fraser F, Hall C, Gill S, Bancroft I: **Six new recombinant inbred populations for the study of quantitative traits in *Arabidopsis thaliana***. *Theor Appl Genet* 2008, **116**: 623-634.

101. Bentolila S, Elliott LE, Hanson MR: **Genetic architecture of mitochondrial editing in *Arabidopsis thaliana***. *Genetics* 2008, **178**: 1693-1708.

102. Teng S, Rognoni S, Bentsink L, Smeekens S: **The Arabidopsis GSQ5/DOG1 Cvi allele is induced by the ABA-mediated sugar signalling pathway, and enhances sugar sensitivity by stimulating ABI4 expression**. *Plant Journal* 2008, **55**: 372-381.

103. Tisne S, Reymond M, Vile D, Fabre J, Dauzat M, Koornneef M, Granier C: **Combined genetic and modeling approaches reveal that epidermal cell area and number in leaves are controlled by leaf and plant developmental processes in Arabidopsis**. *Plant Physiol* 2008, **148**: 1117-1127.

104. Sicard O, Loudet O, Keurentjes JJB, Candresse T, Le Gall O, Revers F, Decroocq V: **Identification of quantitative trait loci controlling symptom development during viral infection in *Arabidopsis thaliana***. *Mol Plant Microbe Interact* 2008, **21**: 198-207.

105. Simon M, Loudet O, Durand S, Berard A, Brunel D, Sennesal FX, Durand-Tardif M, Pelletier G Camilleri C: **Quantitative trait loci mapping in five new large recombinant inbred line populations of *Arabidopsis thaliana* genotyped with consensus single-nucleotide polymorphism markers**. *Genetics* 2008, **178**: 2253-2264.

106. Skrede I, Brochmann C, Borgen L, Rieseberg LH: **Genetics of intrinsic postzygotic isolation in a circumpolar plant species, *Draba Nivalis* (Brassicaceae)**. *Evolution* 2008 **62**: 1840-1851.

107. Kobayashi Y, Kuroda K, Kimura K, Southron-Francis JL, Furuzawa A, Iuchi S, Kobayashi M, Taylor GJ, Koyama H: **Amino acid polymorphisms in strictly conserved domains of a P-type ATPase HMA5 are involved in the mechanism of copper tolerance variation in Arabidopsis**. *Plant physiol*  2008, **148**: 969-980.

108. Alcazar R, Garcia AV, Parker JE, Reymond M: **Incremental steps toward incompatibility revealed by Arabidopsis epistatic interactions modulating salicylic acid pathway activation**. *Proc Natl Acad Sci U S A* 2009, **106**: 334-339.

109. Lin JZ, Ritland K: Quantitative trait loci differentiating the outbreeding *Mimulus guttatus* from the inbreeding *M. platycalyx*. *Genetics* 1997,**146**: 1115-1121.

110. Bradshaw HD, Otto KG, Frewen BE, McKay JK and Schemske DW: Quantitative Trait Loci Affecting Differences in Floral Morphology Between Two Species of Monkeyﬂower (Mimulus). *Genetics* 1998, **149**: 367-382.

111. Kobayashi Y, Koyama H: **QTL analysis of Al tolerance in recombinant inbred lines of *Arabidopsis thaliana***. *Plant Cell Physiol* 2002, **43**: 1526-1533.

112. Ungerer MC, Halldorsdottir SS, Modliszewski JL, Mackay TFC, Purugganan MD: **Quantitative trait loci for inflorescence development in *Arabidopsis thaliana***. *Genetics* 2002, **160**: 1133-1151.

113. Wolyn DJ, Borevitz WO, Loudet O., Schwartz C, Maloof J, Ecker JR, Berry CC, Chory J: **Light-response quantitative trait loci identified with composite interval and eXtreme array mapping in *Arabidopsis thaliana***. *Genetics* 2004,**167**: 907-917.

114. Syed NH Chen J: **Molecular marker genotypes, heterozygosity and genetic interactions explain heterosis in *Arabidopsis thaliana***. *Heredity* 2005, **94**: 295-304.

115. Freeman JS, O'Reilly-Wapstra JM, Vaillancourt RE, Wiggins N, Potts BM: **Quantitative trait loci for key defensive compounds affecting herbivory of eucalypts in Australia**. *New Phytol* 2008, **178**: 846-851.

116. Rowe HC, Hansen BG, Halkier BA, Kleibenstein DJ: **Biochemical networks and epistasis shape the *Arabidopsis thaliana* metabolome**. *Plant cell* 2008, **20**: 1199-1216.
